# Mechanism of HIV-1 Tat RNA translation and its activation by the Tat protein

**DOI:** 10.1186/1742-4690-6-74

**Published:** 2009-08-11

**Authors:** Nicolas Charnay, Roland Ivanyi-Nagy, Ricardo Soto-Rifo, Théophile Ohlmann, Marcelo López-Lastra, Jean-Luc Darlix

**Affiliations:** 1 LaboRetro, Unité de Virologie Humaine INSERM 758, IFR 128, ENS de Lyon, 46 allée d'Italie, 69364 Lyon, France; 2 TEV, Unité de Virologie Humaine INSERM 758, IFR 128, ENS de Lyon, 46 allée d'Italie, 69364 Lyon, France; 3 Laboratorio de Virología Molecular, Centro de Investigaciones Médicas, Facultad de Medicina, Pontificia Universidad Católica de Chile, Marcoleta 391, Santiago, Chile

## Abstract

**Background:**

The human immunodeficiency virus type 1 (HIV-1) Tat protein is a major viral transactivator required for HIV-1 replication. In the nucleus Tat greatly stimulates the synthesis of full-length transcripts from the HIV-1 promoter by causing efficient transcriptional elongation. Tat induces elongation by directly interacting with the bulge of the transactivation response (TAR) RNA, a hairpin-loop located at the 5'-end of all nascent viral transcripts, and by recruiting cellular transcriptional co-activators. In the cytoplasm, Tat is thought to act as a translational activator of HIV-1 mRNAs. Thus, Tat plays a central role in the regulation of HIV-1 gene expression both at the level of mRNA and protein synthesis. The requirement of Tat in these processes poses an essential question on how sufficient amounts of Tat can be made early on in HIV-1 infected cells to sustain its own synthesis. To address this issue we studied translation of the Tat mRNA *in vitro *and in human cells using recombinant monocistronic and dicistronic RNAs containing the 5' untranslated region (5'-UTR) of Tat RNA.

**Results:**

This study shows that the Tat mRNA can be efficiently translated both *in vitro *and in cells. Furthermore, our data suggest that translation initiation from the Tat mRNA probably occurs by a internal ribosome entry site (IRES) mechanism. Finally, we show that Tat protein can strongly stimulate translation from its cognate mRNA in a TAR dependent fashion.

**Conclusion:**

These results indicate that Tat mRNA translation is efficient and benefits from a feedback stimulation by the Tat protein. This translational control mechanism would ensure that minute amounts of Tat mRNA are sufficient to generate enough Tat protein required to stimulate HIV-1 replication.

## Background

The human immunodeficiency virus type 1 (HIV-1) encodes for the three canonical polyprotein precursors Gag, Pol, and Env, which are required for the formation of infectious viral particles by infected cells. In addition, HIV-1 encodes for six regulatory proteins, among which the Tat and the Rev factors are absolutely required for viral gene expression at the transcriptional and post-transcriptional levels in infected cells [1]. HIV-1 Tat is a small basic protein that mainly localizes to the nucleus of infected cells, where it acts as a potent transcriptional activator that is indispensable for the synthesis of the full length viral RNA (reviewed in [2-4]). Transcriptional activation by Tat is mediated by multiple interactions between Tat and the nascent viral TAR RNA and between Tat and cellular factors involved [5] in transcription initiation and elongation such as P-TEFb [4-11]. In addition, Tat has been shown to stimulate translation of viral mRNAs [12-14]. Importantly, this cytoplasmic function of Tat seems to require a nuclear experience, since the RNA-protein complex formed between Tat protein and nuclear factors must be assembled in the nucleus in order to later exert its function in the cytoplasm [12-14]. Thus, the HIV-1 Tat protein plays a central role in the regulation of HIV-1 gene expression both at the level of transcription and protein synthesis. The requirement of Tat in these processes poses an essential question on how sufficient amounts of this viral protein can be made early on in HIV-1 infected cells to sustain its own synthesis. Soon after completion of viral DNA synthesis by reverse transcriptase and before its integration into the host genome, the viral DNA can be transcribed, but this generates only low levels of fully spliced viral mRNAs encoding Tat and Nef [15]. These observations led us to hypothesize that Tat mRNA is translated even under conditions where it is present in minute quantities together with a high concentration of cellular mRNAs.

Translation of mRNA into protein represents an essential step in gene expression. The regulation of translation is a mechanism used to modulate gene expression in a wide range of biological situations including cell growth, development and the response to biological cues or environmental stresses such as viral infection [16-20]. During viral infection at least two general modes of translational control can be envisaged. The first represents a global control, in which the translation of most cellular mRNAs is regulated. This is evident during the infection of some members of the *Picornaviridae *[18-20] where global regulation mainly occurs by the modification of translation initiation factors. The second corresponds to a mRNA-specific control, whereby the translation of a particular mRNA or a defined group of mRNAs is modulated without affecting general protein biosynthesis or the translational status of the cellular transcriptome as a whole. Translational control of a specific mRNA is normally driven by regulatory protein complexes that recognize particular elements that are usually present in the 5' and/or 3' untranslated regions (UTRs) of the target mRNA [21-24]. It is well recognized that translation control of protein synthesis is mostly exerted at the initiation step.

Translation initiation of eukaryotic mRNAs mostly occurs by a scanning mechanism, whereby the 40S ribosomal subunit binds to the mRNA 5' cap structure and scans the RNA in the 5' to 3' direction until an initiation codon in a favourable 'Kozak' context is encountered [25]. Translation initiation involves the recognition of the mRNA 5' cap structure by eIF4F, which is composed of eIF4E, which binds the 5' cap, eIF4A, and eIF4G, which links the mRNA 5' cap (via eIF4E)] and the 40S ribosomal subunit (via eIF3) [26,27]. Studies on picornavirus protein synthesis led to the discovery of an alternative mechanism of translation initiation, via an internal ribosome entry segment (IRES) [28-30]. A major difference between cap-dependent *versus *IRES-mediated ribosome binding and initiation of translation is that the eIF4E component of the eIF4F complex is dispensable for most of the latter activity [31,32]. At present IRESes are defined solely by functional criteria and cannot yet be predicted by the presence of characteristic RNA sequences or structural motifs [30,33]. Despite these apparent experimental restraints, since the initial characterization of IRESes in *Picornaviridae*, viruses from other families including several members of the *Retroviridae *were found to initiate translation via an IRES ([34-41] reviewed in reference [42]). Indeed, internal ribosome entry has been described in alpha- (ASLV), gammaretroviruses (MoMuLV) and lentiviruses (SIV and HIV).

Based on these findings we wanted to study the mechanism by which the Tat mRNA is translated using recombinant monocistronic and bicistronic RNAs containing all or part of the 5' UTR of the Tat mRNA. In addition, we examined the mechanism by which translation of the Tat mRNA is controlled *in vitro *in rabbit reticulocyte lysates (RRL) and in human cells. Our results show that the Tat mRNA is efficiently translated *in vitro *and in cells, despite the presence of large amounts of cellular mRNAs. Moreover, we show that the Tat protein exerts a positive feedback on the translation of its cognate mRNA. Thus, Tat mRNA appears to be efficiently translated even under conditions where it is in minute amounts among highly abundant cellular mRNAs. Taken together our data explains how minute amounts of Tat mRNA can account for viral protein production required to kick-start HIV-1 replication.

## Results

### Molecular cloning of the Tat mRNA sequences

**Figure 1:**
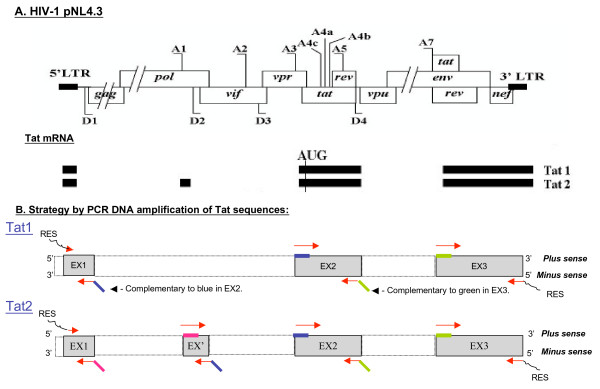
**Reconstitution of the complete Tat RNA sequences**. A. Organization of the splicing donor and acceptor sites in the HIV-1 pNL4.3 genome. B. Reconstitution of the complete Tat1 and Tat2 DNA sequences by PCR. "Hybridization PCR" can associate two different exons. Each Tat1 and Tat2 exon located in the pNL4.3 plasmid sequence (top panel) was independently amplified with specific oligonucleotides (Table 1). In fact, the antisense oligonucleotides used for exon amplification were designed in such a way that their 5' extremity is complementary to the 5' extremity of the next exon sense strand (see the colour codes). With this first PCR, the exon1 sense strand partially hybridizes with the exon2 antisense strand and the exon1 antisense strand with the exon2 sense strand. Then "Amplification PCR" resulted in the accumulation of DNA corresponding to exon1 + exon2. All further steps needed to completely reconstitute the Tat1 and Tat2 sequences were performed using this procedure (Additional file 1). The only difference between Tat1 and Tat2 sequences corresponds to exon EX' (see bottom lane).

**Figure 2:**
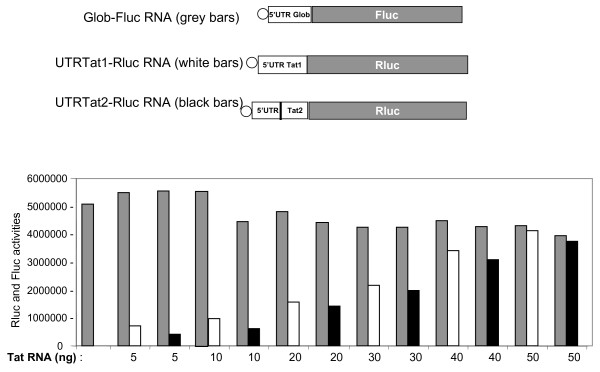
**Translation of Tat1 and Tat2 RNAs in the untreated RRL system**. The top three lanes depict the Glob-Fluc, UTRTat1-Rluc and UTRTat2-Rluc RNAs used in the translation assays in the untreated RRL (URRL). The vertical bar in the 5'UTR of Tat2 represents exon EX' (See fig. 1). Tat1 and Tat2 RNAs were translated in the URRL in the presence of a large excess of endogenous globin mRNA and of *in vitro *generated Glob-Fluc RNA. Independent experiments showed that 50 ng of Glob-Fluc RNA were saturating the URRL. Therefore 50 ng of Glob-Fluc RNA (grey bars) were used per assay together with increasing amounts of UTRTat1-Rluc (white bars) or UTRTat2-Rluc RNA (black bars). Note that under these stringent competition conditions, namely an excess of endogenous globin mRNA as well as Glob-Fluc RNA, UTRTat1/2 RNA at 5 ng (1 × 10^-9 ^M) were well translated (white and black bars, respectively).

**Figure 3:**
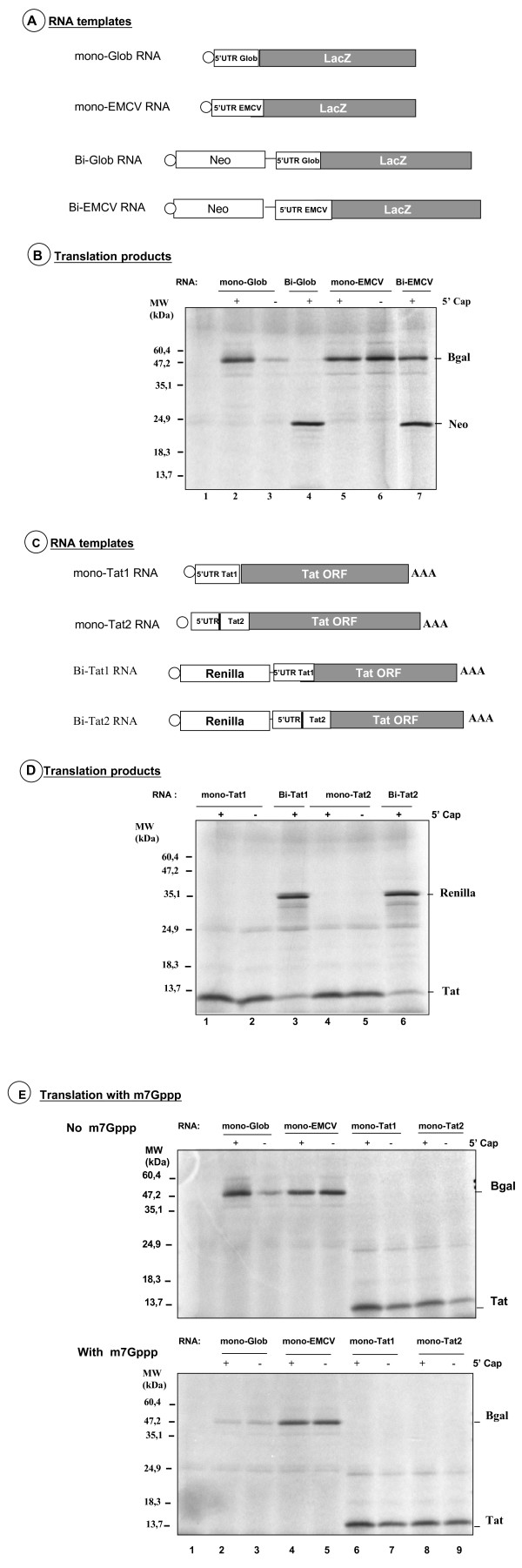
**Translation of the Tat RNA in the RRL system**. A. Structure of the recombinant Glob- and EMCV RNA templates. All recombinant RNAs encode LacZ as the sole gene for the monocistronic RNAs, and as the 3' one for the bicistronic RNAs. The 5' UTR sequences correspond to either the complete 5' leader of the globin mRNA or the EMCV leader (see materials and methods). B. Translation of the mono- and bicistronic RNAs in the RRL system. RNAs were 5' capped (+) or not (-). Note that the non-capped mono-Glob RNA was translated at 15% (lane 3) of the control level (lane 2) while the non-capped mono-EMCV RNA was translated at 125% (lane 6) of the control level (lane 5). LacZ was not translated with the Bi-Glob RNA (lane 4) while it was at 90% (lane 7) the control level with the Bi-EMCV RNA. C. Structure of the recombinant Tat RNAs. The monocistronic and bicistronic recombinant Tat RNAs are shown. D. Translation of the mono- and bicistronic Tat RNAs in the RRL system. Note that mono-Tat1 and mono-Tat2 were translated at the same level either capped (lanes 1 and 4) or non-capped (lanes 2 and 5). Translation of the Tat ORF occurred with the Bi-Tat RNAs, but levels were about 40% (lanes 3 and 6) of the control levels (lanes 1 and 5). E. Translation of the Tat RNAs in the presence of the cap analog 7m-Gppp. Translation conditions were as in B (upper panel), but contained 7m-Gppp (lower panel) during the whole reaction (see methods). Translation of the mono-Glob RNA was extensively inhibited (lane 2) but this was not seen with mono-EMCV RNA (lanes 4 and 5), as expected. Note that translation of the Tat RNAs was not inhibited by the cap analog (compare lanes 6-9 in upper and lower panels).

To study the mechanism of protein synthesis from the Tat mRNA, we cloned the Tat1 and Tat2 sequences, the two major forms of Tat mRNA [43], by means of a PCR-DNA reconstruction protocol (Fig. 1 and Additional file 1). Sequence analysis confirmed that by this strategy (see methods and Fig. 1, panel B; Additional file 1), we were able to fully reconstitute the Tat1 and Tat2 mRNA sequences. Also, we constructed recombinant clones where the Tat1 and Tat2 RNA sequences were inserted next to the Renilla luciferase gene in either a monocistronic or bicistronic vector (Fig 2, 3). To study translation of the Tat RNA, we compared its level of translation with that of a canonical efficiently translated mRNA, namely the globin mRNA, in nuclease treated rabbit reticulocyte lysate (RRL) or untreated RRL (URRL) *in vitro *[44]. In addition, we investigated Tat mRNA translation in HeLa cells, and the impact of the Tat protein on translation of its cognate RNA.

### Tat RNA *versus *globin RNA translation *in vitro*

Soon after its completion the viral DNA can be transcribed by the host cell machinery, but this generates only low levels of fully spliced viral RNAs in the absence of Tat and Rev proteins [15]. To evaluate the efficiency of Tat mRNA translation, we examined the relative translation levels in the RRL of Tat1 and Tat2 RNAs expressing Renilla luciferase (Rluc), in the presence of an excess of Glob-Fluc RNA (Fig. 2), a 5' capped RNA that harbors the 5' UTR of globin mRNA and drives expression of Firefly luciferase (Fluc). Results revealed that the two Tat mRNAs were efficiently translated even in the presence of a high concentration of Glob-Fluc RNA (data not shown). These observations showed that even under unfavorable conditions the Tat mRNA can be efficiently translated. These results prompted us to further characterize the ability of Tat mRNAs to be translated, despite being at a low concentration within a mixture of mRNAs.

The efficiency of Tat RNA translation was studied in the non-nuclease treated RRL (URRL) [44], because it contains a high concentration of endogenous globin mRNA (about 7 × 10^-7 ^M). We examined translation of Tat1 and Tat2 RNAs expressing Rluc in the URRL (Fig. 2A) using conditions where the 5' cap Glob-Fluc was also present in excess (Fig. 2B; grey bars). Results show that under these stringently competitive conditions Tat1 RNA and Tat2 RNA at a concentration of 1 × 10^-9 ^M were translated (Fig. 2B, white and black bars, respectively) and levels of Tat RNA translation linearly increased with increasing RNA concentrations (see white and black bars).

Taken together, these results show that the two HIV-1 Tat RNAs were efficiently translated in the URRL under conditions where both the endogenous globin mRNA and the recombinant Glob Fluc RNA were in vast excess. These findings also show that even at low concentrations the Tat mRNA can efficiently recruit ribosomes for its own translation.

### Investigating Tat RNA translation in the rabbit reticulocyte lysate

The full-length mRNA from gammaretroviruses and lentiviruses can initiate protein synthesis by a cap-independent mechanism ([34,36-40]; reviewed in [42]). In most instances IRESes in retroviruses and retroelements are found within the 5'untranslated region (5'UTR) of the full length mRNA. Furthermore, a report from Brasey et al. [40] suggests that the Tat mRNA would exhibit IRES activity. In order to explore this possibility, translation initiation of the Tat mRNA was studied in the RRL using both monocistronic and bicistronic RNAs. As experimental controls, we used canonical monocistronic and bicistronic RNAs, the translation of which is exclusively 5' cap-dependent (5' UTR of the globin RNA) or cap-independent (5' UTR of EMCV) (Fig. 3A and 3C).

Results reported in figure 3B show that translation in the RRL of the mono 5'Glob-RNA was 5' cap-dependent (see β-galactosidase levels in lanes 2 and 3), while that of the mono EMCV RNA was not (compare lanes 5 and 6). In agreement with this, β-galactosidase was synthesized in the context of the bicistronic Bi-EMCV RNA (lane 7) but clearly not synthesized when the Bi-Glob RNA was used as template (see B-Gal in lane 4). Results showed that for the The Tat RNAs the mono-Tat1 and mono-Tat2 RNAs (Fig 3C) were translated in RRL (Fig. 3D). Strikingly, in the monocistronic context, Tat1 and Tat2 RNA translation occurred independently from the 5' cap structure (Fig. 3D, compare lanes 1–2 and 4–5, respectively). In agreement with this observation, the two cistrons of the Bi-Tat RNAs were clearly expressed in the RRL (see Renilla and Tat in lanes 3 and 6) albeit Tat was synthesized about 2.5 fold less as compared with the monocistronic RNA (compare lanes 2–3 and 5–6 in Fig. 3D). Thus, data show that Tat can be synthesized in a cap-independent manner (Fig. 3C, lanes 1–2) and as the 3' cistron of a bicistronic mRNA (lane 3) while the globin 5' UTR was unable to direct β-galactosidase synthesis under the same experimental conditions (Fig. 3B, lane 4).

To further investigate Tat RNA translation in the RRL, the monocistronic RNAs encoding the Tat protein were translated in the presence of the 7methyl-GTP cap analog (Fig. 3E, bottom panel). The rational of this experiment relies on the competitive binding of initiation factor eIF4E to the m^7^Gppp cap analog, which has been added in excess to the *in vitro *reaction. Figure 3E (top panel, lane 2 and 3) recapitulates results presented in Figure 3B (lanes 2 and 3) where translation of the mono-Glob RNA is cap dependent. As expected, the 7m-GTP cap analog reduced by 7 fold the translation of the capped mono-Glob RNA in the RRL (lanes 2 and 3 in top and bottom panels) and had only a marginal effect on the uncapped mono-Glob RNA. The 7m-GTP cap analog did not affect translation from the mono-EMCV RNA (compare lanes 4–5, in top and bottom panels). In agreement with previous data (Fig. 3D), translation of the mono-Tat RNAs was not altered by the addition of the 7m-GTP cap analog (Fig. 3E, top and bottom panel, lanes 6–9).

Taken together, these results show that in the RRL the HIV-1 Tat mRNA can be translated by an IRES mechanism.

### Tat mRNA translation in human HeLa P4 cells

To examine Tat mRNA translation in cells, we selected the human HeLa P4 cells because this cell line is known to support HIV-1 replication and is a convenient indicator system to monitor HIV-1 Tat-mediated transactivation of the viral LTR. In HeLa P4 cells the expression of the LacZ gene is under the control of the LTR. Therefore, Tat expression will trans-activate the viral LTR and turn on production of β-galactosidase (see methods). In this experimental setting, the expression of the β-galactosidase reporter is used as an indicator of Tat protein production. In a first series of DNA transfection assays, it was found that β-galactosidase was efficiently expressed upon transfection of the full length Tat1 and Tat2 DNAs (data not shown).

**Figure 4:**
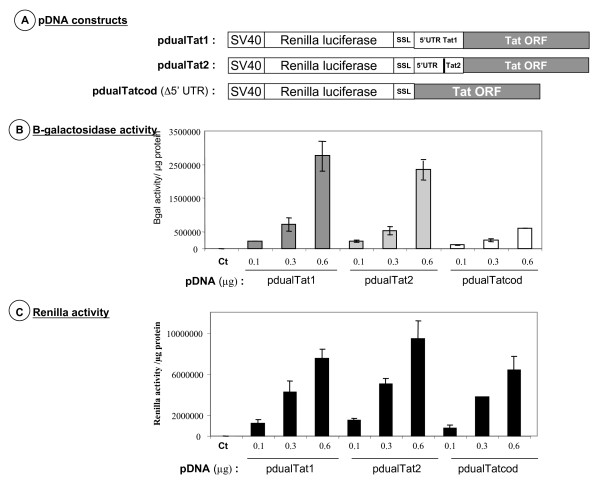
**Expression of Tat in HeLa P4 cells**. A. Top panel depicts the DNA constructs used in the experiments (at least three independent assays were performed). SSL stands for a stable stem-loop to prevent ribosomes translating the Renilla cistron from reading through the Tat coding sequence. The pdualTatcod lacks the 5' UTR of the Tat RNA, except for the 12 nucleotides upstream from the Tat AUG codon (see materials). B. Middle panel shows the activity of newly made Tat, which activates LacZ transcription from the HIV-1 LTR in HeLa P4 cells. This was monitored by the β-galactosidase activity (see methods). C. Lower panel reports the Renilla luciferase activity (5' cistron) for each DNA construct. All values are expressed per μg of total proteins. Integrity of all viral RNA expressed in HeLa P4 cells was assessed by Northern blotting (data not shown).

Next, we constructed bicistronic vectors containing the Renilla luciferase (Rluc) as the cap-dependent 5' cistron and the full length Tat1 or Tat2 sequences as the 3' one (Fig. 4A, pdualTat1 and pdualTat2, respectively). In addition, we constructed a deletion mutant where the Tat 5' UTR was removed; thus this construct contained only the Tat coding sequence with the Tat initiation codon and 12 upstream nucleotides (Fig. 4A, pdualTatcod). In addition a stable stem-loop (SSL) structure was inserted between the two cistrons in order to prevent translating ribosomes from reading through the intercistronic region, thus driving protein synthesis of the second cistron by a termination-reinitiation mechanism [40,45].

Results show that Rluc was expressed in a dose-dependent manner upon transfection of the three recombinant pdual DNAs (Fig. 4C). It is noteworthy that β-galactosidase was expressed at a high level following pdualTat1 and pdualTat2 transfection but was about 5–6 times less with pdualTatcod, the construct lacking the Tat 5'UTR (Fig. 4B). In these experiments, Tat expression from the pdualTatcod vector was considered as background due to the leakiness of the experimental system. These *ex vivo *data support our previous findings indicating that the Tat mRNA can be translated in the context of a bicistronic mRNA by a cap-independent mechanism.

**Figure 5:**
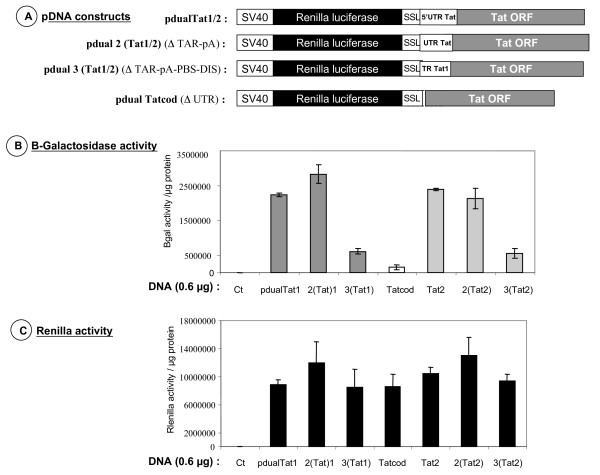
**Expression of 5' UTR mutants of Tat RNA in HeLa P4 cells**. A. Top panel depicts the DNA constructs used in the experiments. Deletions in the 5' UTR of Tat RNA are indicated. B. Middle panel shows the activity of newly made Tat that activates LacZ transcription from the HIV-1 LTR in HeLa P4 cells. This was monitored by the β-galactosidase activity (see methods) (at least three independent assays were performed). Note that deletion of either the entire 5' UTR (Tatcod) or the sequences encompassing TAR-pA-PBS-DIS strongly impaired Tat expression by the bicistronic RNA. C. Lower panel reports the Renilla luciferase activity (5' cistron) for each DNA construct. Integrity of the recombinant RNAs has been examined by Northern blotting. (not shown).

To map sequences essential for Tat RNA translation in such a bicistronic context, we examined the translation of Tat1 and Tat2 recombinant RNAs in which the TAR-polyA (pos. 1–104) and the TAR to the DIS (pos. 1–274) sequences were deleted [see mutants pdual 2(Tat) and pdual 3(Tat) in Fig. 5A]. Results reported in figure 5C show that all vectors expressed Rluc to similar levels. Monitoring Tat production through β-galactosidase activity (Fig. 5B) shows that deletion of the TAR-polyA stem-loops had little influence on Tat expression in HeLa P4 cells, while further deleting the PBS-DIS sequences decreased by 4–5 fold the expression of Tat as evaluated by the level of β-galactosidase activity. As already noted, the expression of the Tat protein from pdualTatcod, which is the negative control, was extremely low (Fig. 5B).

These results indicate that in such a bicistronic context the 5' UTR sequences from the PBS to the Tat initiation codon are necessary for Tat protein synthesis. Taken together, the data presented in figures 3, 4, 5 strongly suggest that the Tat mRNA can be translated via an IRES-dependent mechanism both *in vitro *and in cell culture [40].

### Trans-activation of Tat RNA translation by Tat in HeLa P4 cells

Translational control of specific mRNAs is normally driven by regulatory protein complexes that recognize particular elements that are usually present in the 5' and/or 3' untranslated regions (UTRs) of the target mRNA [21-24]. Because Tat binds with high affinity to the 5' TAR element, we wondered whether such a specific interaction would have an impact on the translation of the Tat mRNAs. Along this line, the Tat-TAR interaction has been described to have an impact on translation of the full length HIV-1 mRNA [13].

**Figure 6:**
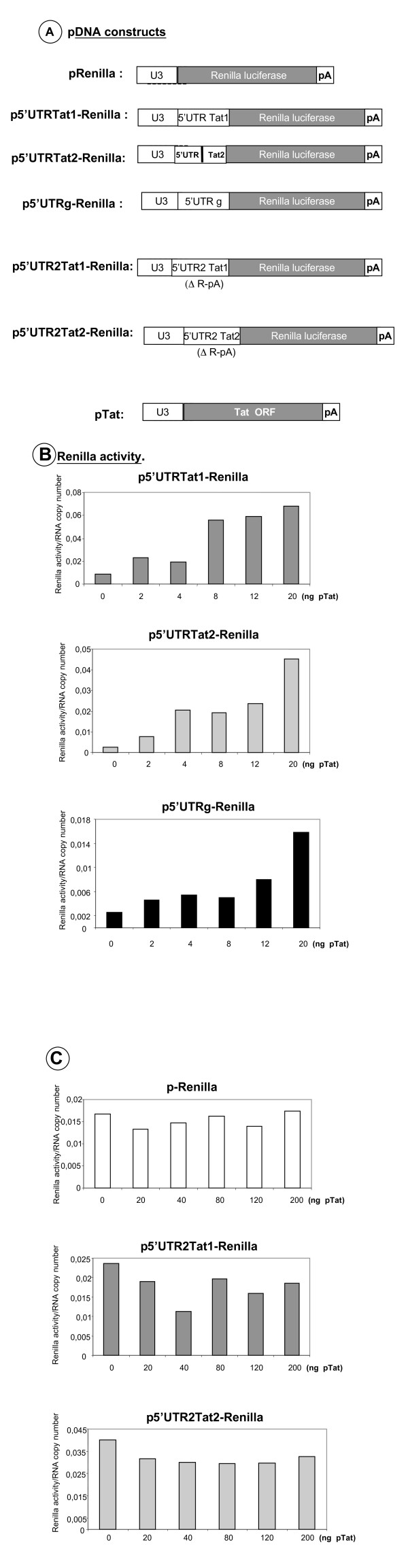
**Tat activates translation of its cognate mRNA**. Panel A shows the monocistronic plasmids encoding Rluc used in these experiments. Panels in B report the results obtained with increasing amounts of pTat DNA, from 0 to 20 ng (at least 3 independent assays were performed). In independent assays, optimal stimulation of Rluc expression was found to occur at 20 ng of pTat (Additional file 2). All results are reported as Rluc activity per RNA copy number monitored by RTqPCR (see methods). Note that the 5' UTR of Tat RNA is more active than that of the genomic RNA with or without pTat addition, in HeLa cells. Panels in C show that Tat did not influence expression of Rluc from plasmid pRenilla (top panel). In addition, the 5' TAR-pA sequences of the 5' UTR of Tat1 or Tat2 appear to be indispensable for Tat-mediated translational activation (lower panels).

To examine this possibility we generated a series of DNA constructs where the Renilla luciferase coding sequence (Rluc) was preceded by a minimal 5' UTR (pRenilla), by the 5' UTR of Tat1 or Tat2 (p5'UTR-Tat Renilla), or by the 5' UTR of the HIV-1 genomic RNA (5' UTR g-Renilla). In addition, we used constructs where the 5' UTR of Tat1 and Tat2 was deleted from the R-U5 sequences (pos. 1–104) (p5'UTR2-Tat Renilla) (Fig. 6A and Additional file 2). The Tat expressing vector contained a minimal 5'UTR followed by the Tat coding sequence (Fig. 6A). Since Tat can strongly activate transcription from the LTR, all Rluc values were normalized to the same copy number of Rluc RNA in HeLa P4 cells, using RT-qPCR (see methods).

Results from a first series of experiments revealed that Tat was able to trans-activate the translation of the UTR-Tat and UTRg-RNAs (Additional file 2B), but not that of pRenilla (data not shown). In the next series of assays, we transfected low quantities of the Tat expressing DNA (from 2 to 20 ng) and monitored Rluc activities (Additional file 2B). Upon normalization to the same RNA copy number as assessed by RT-qPCR (see methods), the results showed that Tat was able to activate by 5–10 fold the translation of the viral Tat RNA and genomic RNA 5' UTRs (see figure 6B and Additional file 2A). This Tat-mediated activation of translation occurred for very low quantities of transfected Tat DNA (2 to 20 ng per 2.5 × 10^5 ^cells), and this was clearly less efficient with higher amounts of Tat DNA (40–200 ng per 2.5 × 10^5 ^cells) (Additional file 2B). It should also be noted that the 5' UTR of the HIV-1 genomic mRNA was about 3–4 fold less efficient than the Tat 5' UTR in promoting Rluc expression in HeLa cells, with or without Tat (Fig. 6B, compare top and bottom panels, first and last bars, respectively).

Interestingly, deletion of the TAR-polyA sequences (p5'UTR2-Tat Renilla) had two effects, leading to a higher level of Rluc translation and no influence of Tat (Fig. 6C) as compared with the p5'UTR-Tat Rluc construct (compare Fig. 6B and 6C). These observations confirm that the HIV-1 5'UTR restricts HIV-1 mRNA translation and suggest that the Tat-TAR interaction relieves the translational repression imposed by the leader structure [13,46,47]. As expected, Tat had no effect on the expression of Rluc using the pRenilla construct (Fig. 6C, top panel).

### Analysis of Tat-mediated activation of Tat RNA translation in the RRL

Several studies show that Tat protein requires other cellular factors to exert the translational activation of the full length HIV-1 mRNA [12-14]. Studies in *Xenopus laevis *oocytes show that the HIV-1 RNA-Tat protein complex must be assembled in the nucleus in order to facilitate translation in the cytoplasm [14]. In agreement with these observations, the above findings show that Tat protein exerts a translational control on viral mRNA translation from the 5'UTR. Furthermore, data show that this phenomenon occurs even when low quantities of the Tat plasmid are used (Fig. 6B). Since Tat has potent RNA binding and chaperoning activities [48] and stimulates translation from the viral mRNA, we sought to evaluate if the Tat-TAR interaction was responsible for the activation of viral RNA translation and to establish if translational control by Tat required other cellular factors. This possibility was investigated *in vitro *in the RRL and URRL using a recombinant version of the Tat (1–86) protein [48], under different experimental conditions.

Firstly, Tat was added to the RRL or URRL followed by either one of the viral RNA, namely UTR-Tat or UTRg-RNA expressing Rluc. Under these conditions, Tat was found to have no, or at best a modest, positive effect on viral RNA translation *in vitro* (data not shown). Secondly, Tat was mixed with the RNA *in vitro*, and then the mix was added to the RRL/URRL translation mixture. Under these conditions translation of RNA containing either the complete 5' UTR of the Tat RNA or of the genomic RNA was decreased up to 3–4 fold upon addition of Tat (Additional file 3). At the same time, Tat only slightly decreased the translation of the Rluc RNA and that of a 5' UTR-Tat RNA where the TAR-polyA has been deleted (Additional file 3). Thirdly, Tat synthesized in the RRL and the Tat/RRL mixture was added to either one of the viral Rluc RNAs and to the control Rluc RNA. Under these conditions, increasing quantities of Tat/RRL were found to strongly inhibit Rluc translation from the viral 5'UTR and only slightly inhibit that of the control Rluc RNA (data not shown).

Taken together these results show that the recombinant Tat protein was not capable of exerting a positive effect on the translation of its cognate mRNA. Furthermore, data suggest that the Tat-TAR interaction inhibited protein synthesis. We therefore reasoned that Tat-mediated translational activation of the HIV-1 RNA might require post-translational modifications [49] and/or cellular cofactors that are absent from the rabbit reticulocyte lysate. To examine this possibility, URLL was supplemented with HeLa cell extracts. The rationale of using these extracts relies on reports showing that HeLa cell extracts support translation of the full length HIV-1 RNA [40] and that supplementation of RRL with cytoplasmic HeLa extracts allowed efficient translation from the HIV-1 genomic 5' UTR [40,50,51]. The addition of increasing amounts of HeLa cell extracts, up to 0.2 μg/μl, to the URRL prior to RNA translation did not modify the pattern of Rluc translation using the viral RNAs or the control RNA. Addition of recombinant Tat (see materials and methods) to the cell extract before translation had a slightly inhibitory effect on viral and control Rluc RNA translation (data not shown).

**Figure 7:**
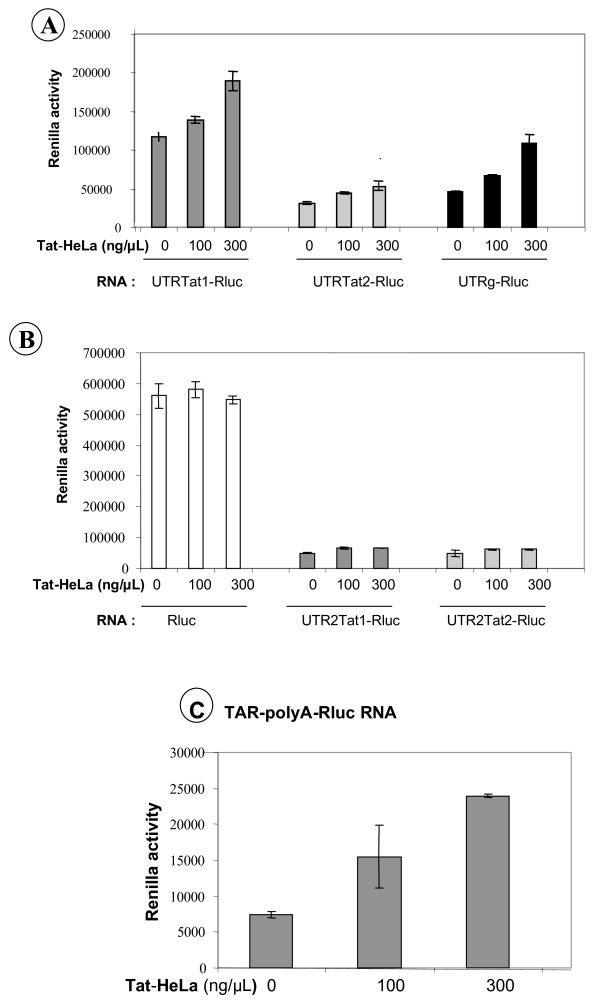
**Influence of Tat-HeLa cell extracts on Tat RNA translation in the RRL**. Tat-HeLa cell extracts were prepared as described in methods and increasing amounts added to the URRL. Results show that addition of increasing amounts of Tat-HeLa cell extracts enhanced translation of the Tat and genomic UTR-Rluc RNA by up to two-fold (Panel A). On the contrary, there was no effect on the translation of Rluc RNA, or of 5' UTR-Rluc RNA lacking the TAR-pA sequences (panel B). In agreement with this, translation of the TAR-pA-Rluc RNA was enhanced by up to three fold by the HeLa-Tat cell extracts (panel C). All experiments were carried out at least three times.

Finally, Tat was transiently expressed to a high level in HeLa cells as assessed by western blotting (see methods and data not shown), and these cells were used to prepare a Tat-HeLa cell extract (see methods). Addition of increasing amounts of the Tat-HeLa extract to the *in vitro *URRL, prior to translation, caused a two fold increase in the level of viral mRNA translation (Fig. 7A), while it had little or no effect on the translation of the control Rluc RNA, or viral RNA deleted from the TAR and polyA structures (Fig. 7B). To further study this Tat-mediated activation of translation *in vitro*, we used a recombinant Rluc RNA where the 5' leader corresponded to the viral 5' TAR-polyA stem-loops. Translation of this recombinant RNA was increased, up to 3 fold, by the Tat-HeLa cell extract (Fig. 7C).

Taken together these results favor the notion that Tat requires post-translational modifications to be fully active as a translational activator of its own mRNA. Alternatively, Tat needs to interact with cellular factors, most probably in the nucleus, in order to be able to activate translation of the HIV-1 Tat and full-length RNAs in the cytoplasm [12-14]. This last possibility stems from the fact that HeLa cell extracts were incapable of assisting Tat-associated translational activation when directly mixed with the viral protein.

## Discussion

**Figure 8:**
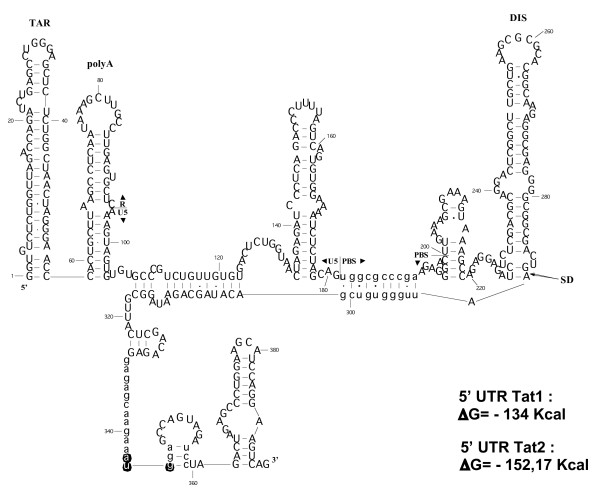
**Computer-assisted folding of the 5' UTR of Tat RNA**. Folding of the 5' UTR of Tat1 RNA was carried out as indicated in methods. Small-case letters indicate that the folding of the given nucleotides was constrained in Mfold (see methods). Sequences and SL structures of importance are indicated, namely from 5' to 3' TAR, polyA (pA), PBS, the SD-SA border, and the Tat AUG (circled). Note that these SLs are present in the secondary structure of the genomic 5' UTR RNA ([39,40,52,55] and ref. therein). Note that the 29-nt long sequence upstream from the Tat initiator AUG is mostly unstructured and thus could serve as a landing pad for the ribosomes. ΔG's for the Tat UTR1 and UTR2, indicated on the right, reveal a high degree of stability (see methods and ref. [52]).

In an attempt to understand how the viral transcriptional factor Tat is initially synthesized for the sustained expression of the viral DNA in newly HIV-1 infected cells, we investigated translation of the Tat mRNAs *in vitro *in rabbit reticulocyte lysate systems (RRL and URRL) and in HeLa cells. Our study focused on the two major forms of the Tat mRNA, namely Tat1 and Tat2 (Fig. 1), because they represent about 80% of all Tat mRNAs in HIV-1 infected cells [43]. Results showed that the HIV-1 Tat mRNA is efficiently translated *in vitro *and in cells to the benefit of Tat synthesis (Fig. 2, 3, 4, 5). The Tat mRNAs possess a 5' cap and a 3' poly(A) tail together with a long 5' leader formed of stable stem-loop structures (Fig. 8). Our data (Fig. 2, 3, 4, 5) indicate that like the full-length HIV-1 mRNA the Tat mRNA can be actively translated by an IRES mechanism [33-40], even when present in low concentrations (Fig. 2). Interestingly, and in accordance with Tat protein function, our data show that low quantities of Tat (Fig 4 and Additional file 2) can transactivate the HIV-1 promoter. The ability of the Tat mRNA to be efficiently translated in combination with the low amount of protein required to transactivate the viral promoter would be essential during the virus replication cycle as Tat protein is absolutely required for the expression of the viral DNA in both its unintegrated and integrated forms [1-3,15].

Tat mRNA translation appears to rely on an IRES mechanism, in a manner similar to that found for the HIV-1 full-length RNA [39,40]. This finding is not without precedent since the Env mRNA of the gammaretrovirus MoMuLV was shown to be translated by an IRES mechanism, in a manner similar to that of the full-length RNA coding for Gag and Gag-Pol [34]. In the case of the HIV-1 genomic IRES, Brasey et al. [40] showed that the IRES overlaps the primer binding site (PBS), the DIS, the splice donor (SD) and the major Psi packaging signal that are located upstream from the Gag initiation codon (Fig. 1 and 8). Evidence was also provided showing that sequences encompassing the PBS, the DIS and the SD had an IRES activity (Figure four in [40]). In agreement with these observations, we found that the TAR and polyA stem-loops were not necessary for the IRES activity of the Tat RNA (Fig. 5). Moreover, the Tat1 IRES activity was found to be clearly more active than its genomic counterpart (Fig. 6).

The above prompted us to look for a possible secondary structure of the 5' UTR of the Tat RNA using a bioinformatic approach (see methods) [52,53]. A putative consensus secondary structure [54] is proposed in figure 8 where the most conserved secondary structures in the 5' UTR of Tat1 and Tat2 RNAs, which are not found in the 5' UTR of the genomic RNA [[55] and ref. therein], are (i) an interaction between the PBS sequence and the 5' part of Tat exon 2, (ii) a small non-structured segment rich in guanine and adenine residues 5' to the Tat AUG codon, and (iii) a small stem-loop next to the Tat AUG codon. In contrast, the TAR and polyA stem-loops remain as individual structures present in the 5' UTR of both the Tat and genomic RNAs, but are not required *per se *for the IRES activity ([40], and Fig. 5). The single-stranded segments present in the Tat 5' UTR could function as a landing pad for the binding of ribosomes near or at the Tat initiator codon (Fig. 8). This possibility is presently under investigation. Moreover, the Tat IRES appears to actively recruit the translation initiation complex to the benefit of Tat synthesis, a process absolutely required for the sustained expression of the viral DNA in the unintegrated or integrated forms [1-3,15]. Cap-dependent translation is suppressed during the G2/M phase of the cell cycle [56]. Interestingly, HIV-1 full length RNA synthesis is highly stimulated during the viral induced G2/M arrest [57]. It is tempting to speculate that this IRES activity ensures Tat protein synthesis during the G2/M phase of the cell cycle [40,58]. Synthesis of Tat during G2/M would be partially responsible for the high degree of transcriptional activity from the viral promoter observed during this phase of the cell cycle.

Next we studied the influence of Tat on the translation of its cognate mRNA. Results showed that Tat was able to trans-activate the translation of Tat RNA, by up to tenfold in HeLa cells (Fig. 6A, B), but not the translation of the Rluc RNA (Fig. 6C, top panel). In addition, the 5' terminal TAR-polyA sequences are required for the activation of Tat RNA translation (Fig. 6C, lower panel). The Tat-mediated activation of translation also most probably benefits the other viral RNAs, notably the genomic RNA (Fig. 6B, lower panel), which is in agreement with the finding of Leibowitz [59]. We did not succeed in fully reconstituting the translational activation of the viral RNAs by Tat in the RRL systems (Fig. 7 and data not shown). A likely explanation for these observations is that the recombinant Tat protein used here conserves the RNA binding activity but is incapable of recruiting cellular proteins required for the Tat-mediated activation of translation. Results obtained with HeLa and Tat-HeLa cell extracts are however in agreement with what has been described in other experimental systems [12-14] and favour the notion that Tat needs to be post-translationally modifed or needs to contact cellular factors in order to be able to activate translation from the viral mRNA. An interesting possibility that also stems from our findings is that Tat expression in cells might transactivate cellular mRNAs coding for proteins required for Tat to function as a translational activator of its cognate mRNA. Tat is a basic protein with nucleic acid binding and chaperoning activities [48], and thus appears to behave as a scaffolding protein for both viral transcription and translation [1-11]. Yet, cellular factors recruited by Tat for the translational activation of the viral RNA remain to be determined [5], and this recruitment is presently under investigation.

## Conclusion

This study shows that the Tat mRNA can be efficiently translated under unfavourable conditions, suggesting that only minute concentrations of mRNA are required to assure the Tat protein concentration needed to stimulate viral mRNA synthesis and translation. Moreover, we show that the viral protein Tat exerts a translational control over its cognate mRNA.

## Methods

### Cell culture

Human HeLa P4 cells, which express the CD4 receptor and the bacterial LacZ gene under the control of the HIV-1 LTR, were maintained in complete Dulbecco's Modified Eagles's Medium with Glutamax (DMEM, Gibco, Life Technologies Corporation, Carlsbad, California, USA), supplemented with 10% FCS and penicillin and streptomycin antibiotics.

### Molecular Biology

#### Construction of the Tat DNA sequences

Because we experienced inaccurate RT-PCR amplification of the Tat mRNAs extracted from infected cells, we decided to construct the DNA fragments representing the Tat1 and Tat2 mRNA sequences by PCR using the HIV-1 pNL4.3 DNA as a template. Specific DNA oligonucleotides (ODNs) (Table 1) used for the PCR reactions were designed according to the Tat splice donor and acceptor sites (D and A, respectively; see fig. 1). For Tat1, RNA splicing removes two introns from D1 (pos. 743 on pNL4.3) to A3 (pos. 5777) and from D4 to A7 (pos. 6044 to 8369). Thus Tat1 RNA is formed of 3 exons in addition to the 5' UTR, namely from pos. 454–743, pos. 5777–6044 and pos. 8369–9528. Tat2 RNA is identical except for an additional very small exon, EX', pos. 4910–4962 (A1-D2; see Fig. 1A).

**Table 1:** DNA oligonucleotides used in the present study

***Oligo DNA***	**Sequence and position on Tat mRNA**
LeaderR sense EcoRI	5' GAATTCGGTCTCTCTGGTTAGACCAGATC 3' (exon1 sense for Tat1 et Tat2: 1→)
Leader rev1	5' TATTCTGCTATGTCGACACCCAATTCAGTCGCCGCCCCTCG 3' (exon1 reverse for Tat1:← 290)
Leader rev2	5' GGATCTCTGCTGTCCCTGCAGTCGCCGCCCCTCG 3' (exon1 reverse for Tat2:← 290)
Ex2Tat sense	5' AATTGGGTGTCGACATAGCAGAATAGGCG 3' (exon2 sense for Tat1:290→ and for Tat2:343→)
Ex2Tat rev	5'GGGATTGGGAGGTGGGTTGCTTTGATAGAGAAGCTTGATGAGTCTGACTG3' (exon2: reverse for Tat1:← 558 and for Tat2:← 611)
Ex'Tat sense	5'CAGGGACAGCAGAGATCCAGTTTGGAAAGGACCAGCAAAGCTCCTCTGGAAAG 3' (exon':sense for Tat2:290→)
Ex'Tat rev	5' CTGCTATGTCGACACCCAATTCTTTCCAGAGGAGCTTTGCTG 3' (exon':reverse for Tat2:← 343)
Ex3Tat sense	5' ACCCACCTCCCAATCCCGAG 3' (exon3: sense for Tat1:558→ and for Tat2: 611→)
Tatrev NotI	5' TAATAATGCGGCCGCAGTACAGGCAAAAAGCAGCTGCTTATATGC 3' (exon3: reverse for Tat1:← 1718 and for Tat2:← 1770)
PBSEcoRI sense	5' TATATTAGAATTCGTGTGCCCGTCTGTTGTGTGACT 3' (exon1: sense for Tat1 and for Tat2:104 →)
SDEcoRI sense	5' ATATAAGAATTCCGAGGGGCGGCGACTG 3' (exon1: sense for Tat1 and for Tat2:274→)
AUGEcoRI sense	5' TATAATAGAATTCATGGAGCCAGTAGATCCTAGACTAGAG 3' (exon2: sense for Tat1:343 → and for Tat2:396 →)
TatNcoI sense	5' TAATATACCATGGGGTCTCTCTGGTTAGACCAGATC 3' (exon1: sense for Tat1 and for Tat2:1→)
TatSmaI rev	5' TATATACCCGGGAGTACAGGCAAAAAGCAGCTGCTTATATGC 3' (exon3: reverse for Tat1:← 1718 and for Tat2:← 1770)
TatXbaI sense	5' ATATATTCTAGAGGTCTCTCTGGTTAGACCAGATC 3' (exon 1:for Tat1 et Tat2:1→)
TatXbaI rev	5' TAATAATTCTAGAAGTACAGGCAAAAAGCAGCTGCTTATATGC 3' (exon3 reverse for Tat1:← 1718 and for Tat2:← 1770)
(111)NcoI rev	5' TATATACCATGGGGCACACACTACTTTGAGCACTCAAGG 3' (exon1:reverse:← 111)
UTRTatNcoI rev	5' TATATTACCATGGTTCTTGCTCTCCTCTGTCGAGTAACG 3' (exon2: reverse for Tat1:← 342 and for Tat2:← 395)
(1-336)NcoI rev	5' ATATATACCATGGCTCTCTCCTTCTAGCCTCCGC 3' (reverse: 5' to AUG of the RNAg)
UTR2TatNcoI sense	5' TAATATACCATGGGTGTGCCCGTCTGTTGTGTGACT 3' (exon1: sense for Tat1 and for Tat2:104 →)
LTRPvuII sense	5' TAATATACAGCTGTGGAAGGGCTAATTTGGTCCC 3' (sense for U3)
LTRPvuII rev	5' TATATTACAGCTGAGTACAGGCAAAAAGCAGCTGC 3' (reverse for U3)

The Tat DNA constructs also contain either the 5' LTR for *ex vivo *expression, or the T7 RNA polymerase promoter for *in vitro *RNA synthesis (see below). In addition, it was necessary to omit the 3' R sequence to prevent frequent recombination reactions during amplification.

The Tat exons were independently PCR amplified (Fig. 1B) using the designed ODNs (Table 1 and Additional file 1) and the Vent polymerase (New England Biolabs, Ipswich, MA, USA). Then, reconstitution of the Tat1 and Tat2 DNA fragments was carried out (Fig. 1B): minus strand ODNs were designed in such a way that their 5' extremity was complementary to the 5' extremity of the plus strand of the next exon (Table 1). Thus, by means of a "hybridization PCR" procedure, each exon was linked to the next one and the resulting DNA was subsequently amplified by PCR using additional ODNs (Table 1).

#### Plasmid DNA construction

(i) Plasmid DNA with Tat sequences. The complete Tat1 and Tat2 DNA fragments (Fig. 1B) and the deleted Tat1 and Tat2 DNA fragments were cloned into pD2EGFP-N1 (Clontech Mountain View, CA, USA) at the EcoRI/NotI sites, in place of the eGFP gene. The Tat DNA fragments in pD2EGFP-N1 were recovered upon EcoRI/XbaI digestion and cloned into the pdualuc bicistronic plasmid [39], cleaved by EcoRI/XbaI.

The same Tat DNA fragments were also cloned into the p0pRenilla vector at the XbaI restriction site, next to the Renilla Luciferase gene.

The HIV-1 U3 promoter/enhancer region was PCR amplified as above using the HIV-1 pNL4.3 DNA template and cloned into p0pRenilla at the PvuII site, generating the pRenilla plasmid. We used this new DNA construct to insert at the NcoI site, 5' to the Renilla gene, each one of the DNA fragments corresponding to the complete 5' UTR of Tat1 and Tat2 RNAs, to the deleted 5' UTR (delta R-U5) of Tat1 and Tat2 RNAs, to the 5' UTR of the viral genomic RNA, and to the 5' first 111 nt of Tat RNA.

The Tat1 and Tat2 DNA fragments were also cloned into pRenilla at the NcoI/SmaI sites, in place of the Renilla Luciferase gene.

(ii) Other plasmid DNA. Plasmids pAB300-UTRGlobin and pAB300-UTREMCV contain the 5' UTR of the globin and EMCV RNA, respectively, at the NheI site just before the LacZ gene in pAB300.

Plasmids pBis-UTRGlobin and pBis-UTREMCV contain the 5' UTR of the globin and EMCV RNA, respectively, at the NheI site in the intercistronic region of the pBis plasmid [40].

#### DNA transfection

HeLaP4 cells were plated at 250 000 cells per well in six-well plates in complete medium and DNA was transfected using Lipofectamine and Plus reagent (Invitrogen Life Technologies Corporation, Carlsbad, California, USA). 48 hours post transfection, HeLa P4 cells were lysed with 250 μl of lysis buffer per well and the Renilla Luciferase and β-Galactosidase activities were monitored with the 'Renilla Luciferase Assay system' (Promega Corporation, Madison, WI, USA) and 'β-gal Reporter Gene Assay' (Roche Molecular Systems, INC., Branchburg, NJ, USA), respectively. All measurements were performed with a Promega luminometer by substrate injection. All results were normalized for the same amount of total proteins in the HeLa cell extracts.

#### Reverse transcription and quantitative PCR reactions

RNA was extracted from cells using the Trizol reagent (Invitrogen), according to the manufacturer's instructions. Two μg of total cellular RNA were used per reverse transcription reaction using the SuperScript II reverse transcriptase (Invitrogen), and 1 μM of the given ODN (Table 1).

The mixture was heated for 5 min at 65°C and then kept on ice. Next it was incubated for 2 min at 42°C and RT was added. The reaction was for 50 min at 42°C.

To quantitatively assess RNA levels by cDNA amplification, we used the 'LighCycler FastStart DNA Master SYBR Green kit (Roche).

### *In vitro *RNA synthesis and translation

#### *In vitro *transcription

The DNA templates of interest were linearized, purified by a phenol/chloroform extraction, and ethanol precipitated. 2 μg of DNAs were used per transcription reaction. *In vitro *RNA synthesis was performed as previously described [38,44] for 1 h 30 min at 37°C in 50 μl final volume. For capped RNA synthesis, m7Gppp was added at the beginning of the reaction at 1 mM final concentration.

#### RNA recovery

40 μl of LiCl (7.5 M, 75 mM EDTA) were added to the transcription reaction, which was kept for 30 min at -20°C. Then RNAs were recovered by centrifugation at 14000 g, 4°C for 30 min. The RNA pellets were washed with 120 μl of 70% ethanol, dissolved in 30 μl of pure water and kept at -20°C.

#### RNA translation in the rabbit reticulocyte lysate system

*In vitro *synthesized RNAs (5–100 ng) were translated in 10 μl of either 25% Flexi^® ^Rabbit Reticulocyte System (Promega, USA) or the supplemented untreated RRL 50% (v/v) (as described in [44]) in the presence of 75 mM KCl, 0.5 mM MgCl_2_, 20 μM of each amino acid (minus cysteine) and 0.6 mCi/ml of [^35^S]-cystein (GE Healthcare Life Sciences Piscataway, NJ, USA).

Tat protein added to the *in vitro *translation reactions was chemically synthesized as described in [48] (CNRS, Immunologie et Chimie Thérapeutiques, UPR 9021- Strasbourg).

Reactions were at 30°C for 45 min and stopped by the addition of 90 μl of buffer (0.1 mM DTT; 35% glycerol; 0.2 M Tris-HCl pH 6.8; 1% SDS; 0.5% bromophenol blue). 10 μl were loaded onto a 15% polyacrylamide-SDS gel (PAGE-SDS). After protein resolution, the gel was fixed in a solution containing 30% methanol and 10% acetic acid for 30 min, and subjected to autoradiography using Biomax films (Eastman Kodak, USA). Densitometric analyses were performed by Phospho Imaging with a Storm 850 phosphoimager. To evaluate the translation level of RNA encoding Renilla Luciferase, we monitored the Renilla luciferase activity directly from the translation reaction. Reactions were stopped with 40 μl of lysis buffer from the " Renilla Luciferase assay system" and 20 μl of that mixture were used to quantify the Renilla luciferase activity by luminometry.

#### Cytoplasmic extract of HeLa P4 cells

HeLa P4 cells (1 × 10^7^) were washed with PBS, trypsinized and transferred in a 15 ml tube, and then centrifuged at 1500 rpm at 4°C for 5 min. All subsequent steps were carried out on ice. The cell pellet was washed twice with 10 ml of PBS, 2% FCS and centrifuged at 1500 rpm for 5 min. The cell pellet was resuspended in two volumes of hypotonic buffer (HEPES-KOH 10 mM, pH 7.6, potassium acetate 10 mM, MgOAc 0.5 mM, DTT 1 mM, protease inhibitors, and RNasin (40 U/ml)), and cells were lysed by passing through a needle. The cellular lysate was centrifuged at 14000 g for 10 min and the supernatant was analysed for its total protein content and kept at -80°C.

#### Western blotting

Two methods have been used to directly assess Tat expression in HeLa P4 cells.

Firstly, a Hybond-P membrane (GE Healthcare) was activated by means of a methanol-air treatment and rinsed in 20% methanol, 25 mM Tris pH 8 and 192 mM Glycine. Then, 0.5, 1, 2 and 5 μg of total or cytoplasmic extracts from HeLa P4 cells expressing HIV-1 Tat were carefully spotted onto the membrane. Next, the membrane was incubated during 1 h at 20°C in TBS-T (Tris-HCl pH 8, 50 mM, NaCl 0.15 M, 0.5% Tween 20) containing 5% milk powder, and then for 12–14 hours at 4°C in the presence of the mouse monoclonal anti-Tat antibody (antiTat7S directed againts the basic region of Tat) (a kind gift from Michel Leonetti, the CEA, France). Then, the membrane was extensively rinsed three times in TBS-T, and incubated with an anti-mouse IgG antibody (Dako). The membrane was rinsed three times in TBS-T, and incubated 5 min in the presence of the peroxydase substrate (Supersignal West Pico Chemiluminescent kit, Perbio).

Secondly, classical western blotting was carried out as above except that the cellular extracts (15 μg) were run over a 15% SDS PAGE gel. Detection of the chemoluminescent signals was carried out by autoradiography, as before.

#### Bioinformatics

A putative secondary structure for the 5' UTR of Tat1 and Tat2 RNAs was determined by means of bioinformatic analyses.

Firstly, 27 divergent sequences were selected in the HIV-1 genomic RNA database (Los Alamos database) . Reconstitution of the complete Tat1 and Tat2 RNA sequences was carried out by manual splicing. Then, the sequences were aligned with the ClustalW software [53], and the alignments were manually edited. The obtained alignments were used to establish a consensus secondary structure for the Tat1 and Tat2 RNAs, using the RNAalifold software of the Vienna RNA package [52]. Variability in the aligned RNA sequences, with special emphasis on co-variant sites, together with the consensus structure, was used to infer putatively conserved secondary structures in Tat1 and Tat2 RNAs. Apart from the TAR stem-loop, the most conserved structural features in the 5' UTR of Tat RNAs are: *(i) *an interaction between the PBS sequence and the 5' part of Tat1 exon 2 which is conserved in all sequences of the Los Alamos database; *(ii) *a small, non-structured segment rich in guanine and adenine 5' to the Tat AUG codon; and *(iii) *a small stem-loop next to the Tat AUG codon (Fig. 8). These features were used as constraints in Mfold [54] for the folding of the pNL4.3 Tat RNA sequences.

## Competing interests

The authors declare that they have no competing interests.

## Authors' contributions

NC performed in vitro and ex vivo experiments. RIN analyzed the Tat mRNA 5' UTR structure and corrected the manuscript RSR contributed to the study design and constructed some of the plasmids used in this study. TO and MLL assisted with manuscript preparation and edition. JLD contributed to the study design and wrote the manuscript

## Supplementary Material

Additional file 1 **Supplementary Figure S1**. the basic hybridization and amplification PCR protocols to reconstitute the Tat1 and Tat2 mRNAs. Click here for file

Additional file 2 **Supplementary Figure S2. Tat activates translation of its own mRNA. Plasmid constructs are shown in figure **6A. Figure A reports the influence of increasing amounts of pTat DNA on the Renilla activity of p5'UTRTat1-Renilla, p5'UTRTat2-Renilla and p5'UTRg-Renilla constructs. Renilla activities per RNA copy number are shown in figure 6B. Figure B reports the results obtained with increasing amounts of pTat DNA, from 0 to 200 ng (at least 3 independent assays were performed). All results are reported as Rluc activity per RNA copy number (see methods). Click here for file

Additional file 3 **Supplementary Figure S3. Influence of Tat protein on RNA translation in the RRL. Structures of the RNA templates are described in materials and in figure **2. The Tat (1–86) protein was provided by S. Muller (CNRS, Strasbourg) and was bound to the relevant RNA template (see figure) before translation in the RRL. Binding of Tat caused a translation inhibition of the viral RNAs containing the complete 5' UTR (panel A) and much less inhibition of Rluc RNA and viral RNAs missing the TAR-pA sequences (panel B). Click here for file
