# Supplementary material for: Mechanism of HIV-1 Tat RNA translation and its activation by the Tat protein
Source: Retrovirology. 2009 Aug 11;6:74. doi: 10.1186/1742-4690-6-74 (PMC2739156; doi:10.1186/1742-4690-6-74)
Supplement: Additional file 1 — Supplementary Figure S1. the basic hybridization and amplification PCR protocols to reconstitute the Tat1 and Tat2 mRNAs. [file 1742-4690-6-74-S1.ppt]

## Slide 1
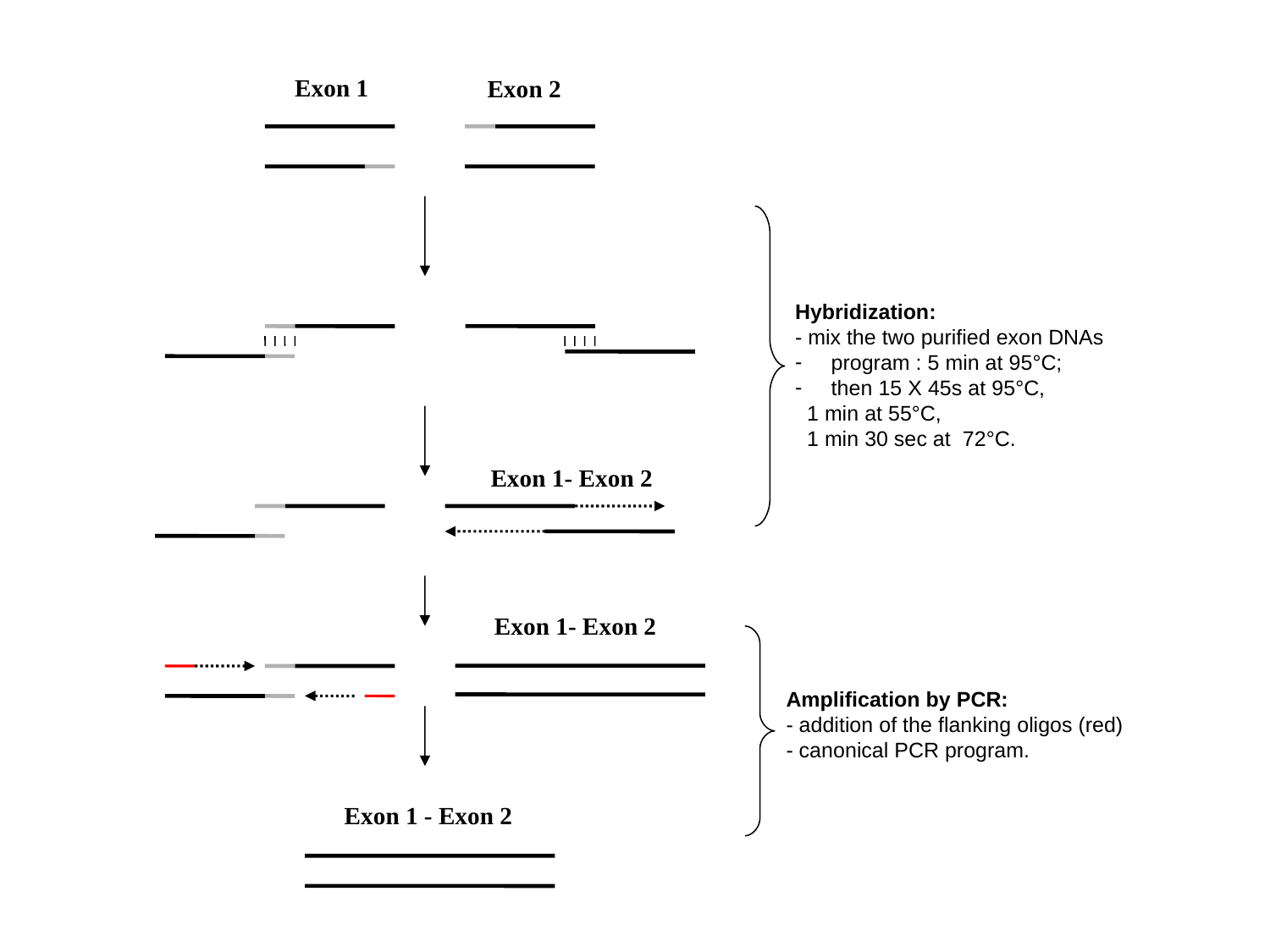

Exon 1
Exon 2
Hybridization:
- mix the two purified exon DNAs
 program : 5 min at 95°C;
 then 15 X 45s at 95°C,
 1 min at 55°C,
 1 min 30 sec at 72°C.
Exon 1- Exon 2
Exon 1- Exon 2
Amplification by PCR:
- addition of the flanking oligos (red)
- canonical PCR program.
Exon 1 - Exon 2
