# Supplementary material for: Mechanism of HIV-1 Tat RNA translation and its activation by the Tat protein
Source: Retrovirology. 2009 Aug 11;6:74. doi: 10.1186/1742-4690-6-74 (PMC2739156; doi:10.1186/1742-4690-6-74)
Supplement: Additional file 2 — Supplementary Figure S2. Tat activates translation of its own mRNA. Plasmid constructs are shown in figure 6A. Figure A reports the influence of increasing amounts of pTat DNA on the Renilla activity of p5'UTRTat1-Renilla, p5'UTRTat2-Renilla and p5'UTRg-Renilla constructs. Renilla activities per RNA copy number are shown in figure 6B. Figure B reports the results obtained with increasing amounts of pTat DNA, from 0 to 200 ng (at least 3 independent assays were performed). All results are reported as Rluc activity per RNA copy number (see methods). [file 1742-4690-6-74-S2.ppt]

## Slide 1
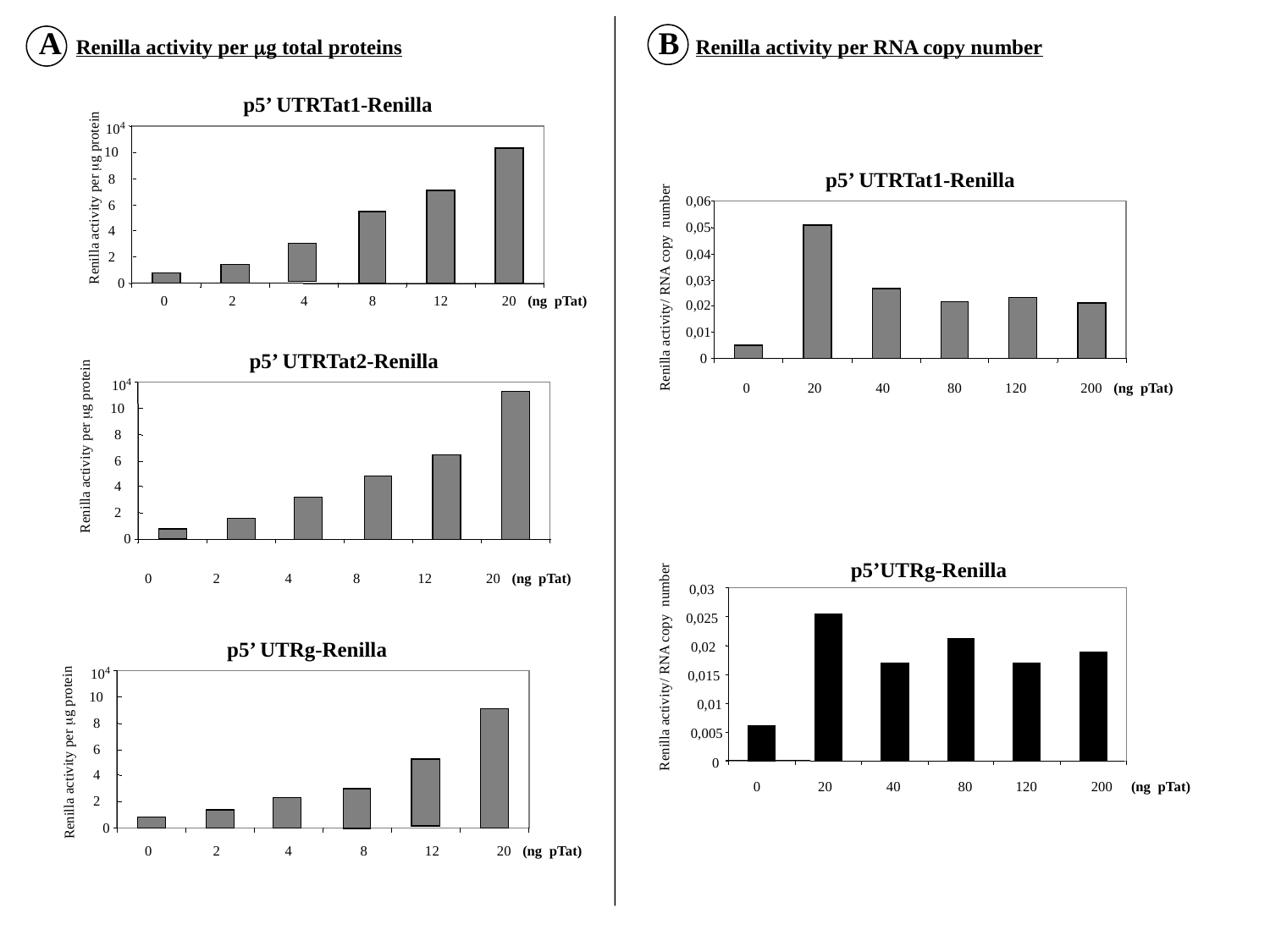

A Renilla activity per μg total proteins
B Renilla activity per RNA copy number
p5’ UTRTat1-Renilla
104
10
p5’ UTRTat1-Renilla
8
Renilla activity per μg protein
0,06
6
0,05
4
0,04
2
0,03
0
Renilla activity/ RNA copy number
 0 2 4 8 12 20 (ng pTat)
0,02
0,01
p5’ UTRTat2-Renilla
0
 0 20 40 80 120 200 (ng pTat)
104
10
8
Renilla activity per μg protein
6
4
2
0
p5’UTRg-Renilla
 0 2 4 8 12 20 (ng pTat)
0,03
0,025
p5’ UTRg-Renilla
0,02
Renilla activity/ RNA copy number
104
0,015
10
0,01
8
0,005
6
Renilla activity per μg protein
0
4
0 20 40 80 120 200 (ng pTat)
2
0
 0 2 4 8 12 20 (ng pTat)
