# Supplementary material for: Mechanism of HIV-1 Tat RNA translation and its activation by the Tat protein
Source: Retrovirology. 2009 Aug 11;6:74. doi: 10.1186/1742-4690-6-74 (PMC2739156; doi:10.1186/1742-4690-6-74)
Supplement: Additional file 3 — Supplementary Figure S3. Influence of Tat protein on RNA translation in the RRL. Structures of the RNA templates are described in materials and in figure 2. The Tat (1–86) protein was provided by S. Muller (CNRS, Strasbourg) and was bound to the relevant RNA template (see figure) before translation in the RRL. Binding of Tat caused a translation inhibition of the viral RNAs containing the complete 5' UTR (panel A) and much less inhibition of Rluc RNA and viral RNAs missing the TAR-pA sequences (panel B). [file 1742-4690-6-74-S3.ppt]

## Slide 1
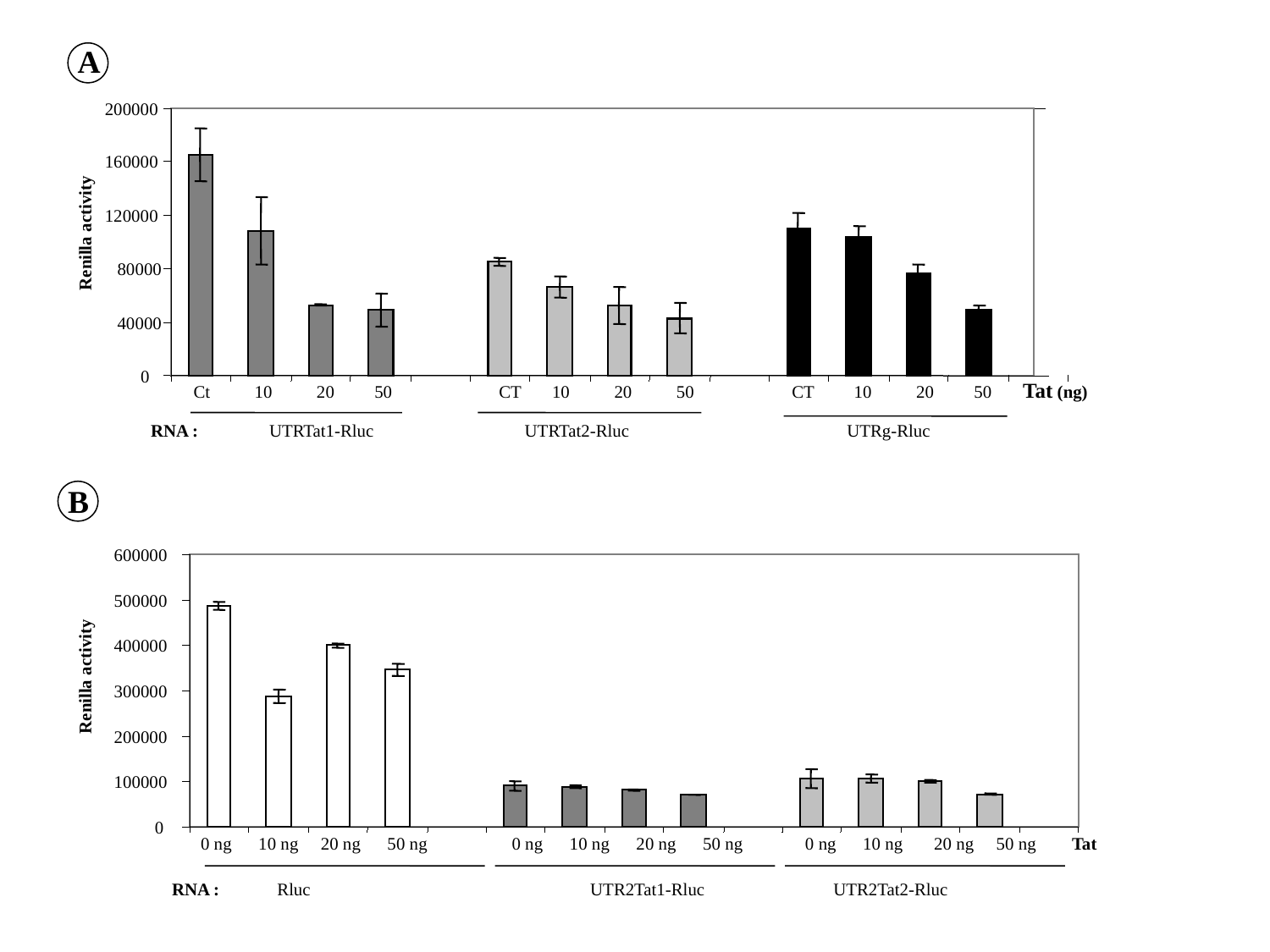

A
200000
160000
120000
Renilla activity
80000
40000
0
 Ct 10 20 50 CT 10 20 50 CT 10 20 50 Tat (ng)
 RNA : UTRTat1-Rluc UTRTat2-Rluc UTRg-Rluc
B
600000
500000
400000
Renilla activity
300000
200000
100000
0
 0 ng 10 ng 20 ng 50 ng 0 ng 10 ng 20 ng 50 ng 0 ng 10 ng 20 ng 50 ng Tat
 RNA : Rluc UTR2Tat1-Rluc UTR2Tat2-Rluc
